# Overexpression of pyruvate dehydrogenase phosphatase 1 promotes the progression of pancreatic adenocarcinoma by regulating energy-related AMPK/mTOR signaling

**DOI:** 10.1186/s13578-020-00457-5

**Published:** 2020-08-06

**Authors:** Ye Li, Jia Shen, Chien-shan Cheng, HuiFeng Gao, Jiangang Zhao, Lianyu Chen

**Affiliations:** 1 Department of Integrated Oncology, Fudan University Shanghai Cancer Center, Shanghai, 200032 China; 2 Department of Oncology, Shanghai Medical College, Fudan University, Shanghai, 200032 China; 3 Department of Oncology, First People’s Hospital of Pinghu, Zhejiang, 314200 China; 4 Department of Oncology, Shaoxing Central Hospital, Zhejiang, 312030 China

**Keywords:** Pancreatic adenocarcinoma, Pyruvate dehydrogenase phosphatase 1, ATP, mTOR, AMPK

## Abstract

**Background:**

Human pyruvate dehydrogenase phosphatase 1 (PDP1) plays an important physiological role in energy metabolism; however, its expression and function in human pancreatic adenocarcinoma (PDAC) remain unknown. This study aimed to investigate the expression pattern and mechanisms of action of PDP1 in human PDAC.

**Methods:**

The expression pattern of PDP1 in PDAC was determined, and its correlation with patient survival was analyzed. Ectopic expression or knockdown of PDP1 was performed, and in vitro proliferation and migration, as well as in vivo tumor growth of PDAC, were measured. The mechanism was studied by biochemical approaches.

**Results:**

PDP1 was overexpressed in human PDAC samples, and high expression of PDP1 correlated with poor overall and disease-free survival of PDAC patients. PDP1 promoted the proliferation, colony formation, and invasion of PDAC cells in vitro and facilitated orthotopic tumor growth in vivo. PDP1 accelerated intracellular ATP production, leading to sufficient energy to support rapid cancer progression. mTOR activation was responsible for the PDP1-induced tumor cell proliferation and invasion in PDAC. AMPK was downregulated by PDP1 overexpression, resulting in mTOR activation and cancer progression.

**Conclusion:**

Our findings suggested that PDP1 could be a promising diagnostic and therapeutic target for anti-PDAC treatment.

## Introduction

Pancreatic adenocarcinoma (PDAC), which accounts for 90% of pancreatic cancer cases, is among the top 10 life-threatening cancers, with a very high death rate and a survival rate of only approximately 5% [1]. The malignancy of PDAC has been increasing in recent years, and this disease is estimated to be the second leading cause of cancer-related death in the USA [2]. The dismal prognosis of PDAC could be due to the lack of sensitive detection at its early stage, although many biomarkers have been suggested as indicators of PDAC in various experimental studies [3]. Effective treatments for PDAC are unavailable. Although surgical resection is recommended as the only curative treatment [4], only a very low number of patients who have small tumors that are detected early are suitable for surgery [5]. Chemotherapy as the first-line treatment is applied to nonsurgical PDAC patients [6]; however, the response rate is low, and these drugs can only prolong survival by a few months, even in responding patients [7]. Identifying new prognostic and therapeutic biomarkers for PDAC patients is important.

The human PDP1 gene encodes pyruvate dehydrogenase phosphatase (PDP) 1, one of the two PDP isoforms in mammalian cells [8]. Physiologically, PDP1 serves as a critical regulator of the pyruvate dehydrogenases complex (PDC); PDP1 positively regulates the catalytic activities of PDC by the removal of phosphates from the serine sites on E1α of the complex [9]. Upon activation by PDP1, the PDC irreversibly triggers oxidative decarboxylation of pyruvate into acetyl-CoA, which is the main substrate for cellular energy production [10]. Mutation of PDP1 in some patients may cause PDC deficiency, a genetic disorder characterized by neurodegeneration and abnormal metabolism [11]. Loss of PDP1 may also cause intolerance to exercise and mild developmental delay in patients [8, 12] and may produce a lethal phenotype in infants [13]. The expression of PDP1 in muscle cells of obese and diabetic subjects was reduced and could be reversed by endurance training [14, 15]. The loss of PDP1 in obesity was found to signal insulin resistance that could be reversed by plasma insulin supplementation [16]. Overexpression of PDP1 was also found in human prostate cancer and could promote cell proliferation and tumor growth [10]. The expression pattern and function of PDP1 in PDAC remain unclear.

In this study, we attempted to identify the expression and functional role of PDP1 in human PDAC. The expression pattern of PDP1 in human PDAC samples was illustrated by extracting data from the GEO database. The correlation between PDP1 expression and patient survival was profiled to evaluate its prognostic value. The functional role of PDP1 in the proliferation, growth, and invasion of PDAC cells was then evaluated in cellular and animal models. The signaling pathway involved in the regulation of PDP1 was elucidated. We believe this study will shed light on the prognostic and therapeutic value of PDP1 in PDAC.

## Materials and methods

### Reagents, plasmids, and antibodies

Sodium acetate and compound C were obtained from Sigma-Aldrich (USA). Human and murine ORF clones of PDP1 and shRNA against human PDP1 were purchased from Origene (USA). Antibodies against PDP1 (#65575), phospho-mTOR (#2974), mTOR (#2983), phospho-AMPKα (#2535), AMPKα (#5831), PCNA (#2586) and β-actin (#4970) were purchased from Cell Signaling Technologies (USA).

### Cell line and cell culture

The PDAC cell line KP3 was purchased from the Japanese Collection of Research Bioresources Cell Bank. Cells were cultured in RPMI 1640 medium supplemented with 10% fetal bovine serum (FBS) and 1% penicillin/streptomycin under humid conditions of 5% CO_2_ and 37 °C. Panc2 cells were obtained from the Frederick National Laboratory for Cancer Research (Frederick, MD, USA). Cells were cultured in DMEM supplemented with 10% FBS and 1% penicillin/streptomycin under humid conditions of 5% CO_2_ and 37 °C.

### Animal study

The animal study was performed in accordance with the approved protocol by Fudan University (Ref. No.: 2019-FUSCC-JS-017). In brief, Panc2 cells tagged with a luciferase reporter were mixed with Matrigel Matrix (BD Bioscience, USA). A 20 μl mixture containing 1 × 10^8^ Panc2 cells was injected into the pancreas of C57/BL/J mice. Measurement of orthotopic tumor size was performed once per week from 1 week post-injection, using the IVIS Spectrum live animal imager (Perkin-Elmer, USA) with luciferin (30 mg/kg, i.p.) as a substrate. At the end of the study, the mice were sacrificed, and the pancreas was dissected.

### Cell proliferation and colony formation assays

Cell proliferation was measured by cell counting. In brief, 10,000 cells were seeded for growth and were counted at 3, 6 and 9 days of cultivation. The formation of colonies was measured by colony formation assays. A total of 10,000 cells were seeded for growth for 12 days. Cells were fixed with 4% paraformaldehyde and stained with 2% crystal violet. The number of colonies formed was counted.

### Cell invasion assay

The invasiveness of PDAC cells was measured by chamber assays. In brief, 200,000 cells were seeded onto the upper chamber of inserts precoated with Matrigel matrix (BD Bioscience, USA). The receiving chamber was filled with DMEM supplemented with 10% FBS. Forty-eight hours later, the medium on the upper chamber was removed, and cells that migrated through the Matrigel matrix were dissociated with calcein-AM-containing buffer. The fluorescence intensity, representing the number of invaded cells, was measured by a fluorescence microplate reader.

### Immunoblotting

Total protein was extracted from the cell pellets using RIPA buffer and separated by SDS-PAGE. Protein was then transferred onto the PDVF membrane, which was blocked in 5% BSA blocking buffer for 2 h at room temperature. Then, the membrane was incubated with primary antibodies overnight at 4 °C followed by secondary antibody incubation for 2 h at room temperature. The membrane was visualized with chemiluminescence (Bio-Rad, USA) using ECL Select as a substrate (GE Healthcare, Germany).

### Intracellular ATP measurement

Intracellular ATP measurement was performed with a commercial kit (Biovision, USA). In brief, 1 × 106 cells were lysed with 100 μl of ATP assay buffer, followed by deproteinization through a 10 kDa spin column. Then, 50 μl of flow through was mixed with 44 μl of ATP assay buffer, 2 μl of ATP probe, 2 μl of ATP converter, and 2 μl of developmental reagent for reaction in the dark for 30 min at room temperature. The absorbance was then read at 570 nm. The concentration of ATP in the cell samples was calculated with a standard curve plotted using an ATP standard.

### Histology

Paraffin-embedded pancreatic sections were prepared, and 5-mm slices were generated. For histological measurement of PDAC, the slides were stained with hematoxylin & eosin (H&E). The image was captured under a light microscope.

### Statistical analysis

Data are presented as the mean ± S.D. Data were analyzed by one-way ANOVA, and p < 0.05 was considered statistically significant. All experiments were performed in triplicate unless stated otherwise.

## Results

### PDP1 was overexpressed in PDAC and overexpression of PDP1 predicted poor prognosis of patients

**Fig. 1:**
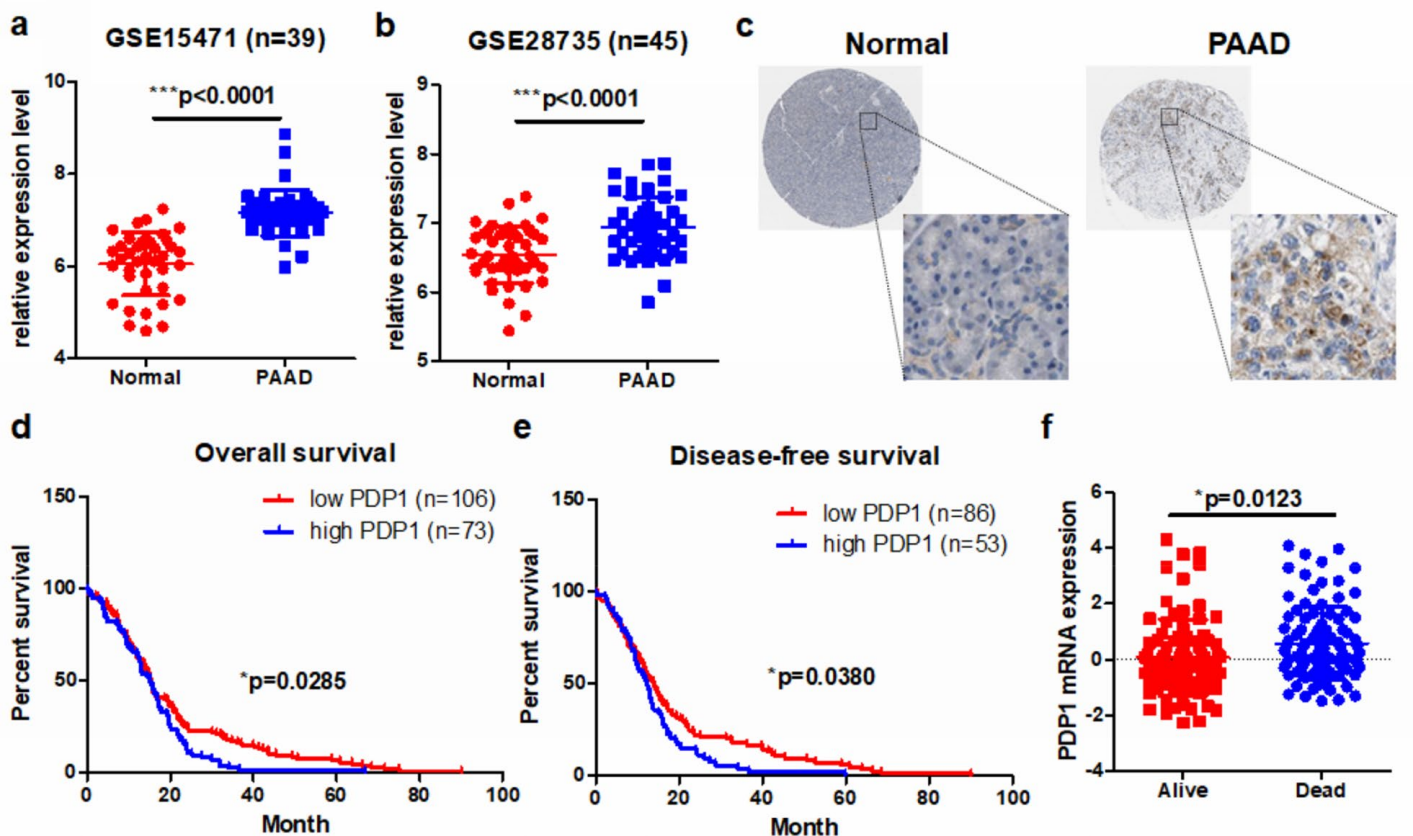
PDP1 was overexpressed in PDAC. Data were retrieved from the GEO database, and PDP1 was found to be overexpressed in PDAC tissues compared with normal pancreatic tissues in (**a**) GSE15471 and (**b**) GSE28735. **c** Immunohistochemical staining of PDP1 was retrieved from the Human Protein Atlas, which suggested that PDP1 protein expression was upregulated in PDAC tissues. Survival of PDAC patients was retrieved from TGCA database, and patients with PDP1 expression above the median level had poor (**d**) overall survival and (**e**) disease-free survival. **f** Surviving PDAC patients had lower expression of PDP1 than the censored patients

The expression and function of PDP1 in PDAC have not been reported. A recent study showed that the expression of PDP1 was elevated in prostate cancer, in which ectopic expression of PDP1 promoted cancer growth and progression [10]. Another study, however, observed that phosphorylation of PDP1 at Tyr94 suppressed its activity and reduced tumor growth [17]. These divergent observations indicated that PDP1 may have different functions in particular types of cancers. To determine the role of PDP1 in PDAC, we first examined its expression in human PDAC samples. Data retrieved from two published datasets of PDAC, GSE15471 and GSE28735, revealed that PDP1 was overexpressed in human PDAC tissues compared with the nonmalignant normal pancreatic tissues (Fig. 1a, b). Immunohistochemical examination of the tissue blocks confirmed that the protein expression of PDP1 was substantially elevated in the PDAC tissues (Fig. 1c). To determine the clinical significance of this elevation, we retrieved patient survival data from TCGA database. We found that the expression of PDP1 predicted poor overall survival and disease-free survival of the PDAC patients (p = 0.0285 and p = 0.0380, respectively), which indicates that PDP1 may be a poor prognostic factor in PDAC (Fig. 1d, e). Furthermore, PDP1 may be a marker for patient mortality, as its expression was significantly higher in patients who died than those who survived (p = 0.0123, Fig. 1f). These findings suggested that PDP1 was overexpressed in PDAC tissues and its overexpression can predict a poor prognosis for patients.

### PDP1 regulates the in vitro proliferation and invasion of PDAC cells

**Fig. 2:**
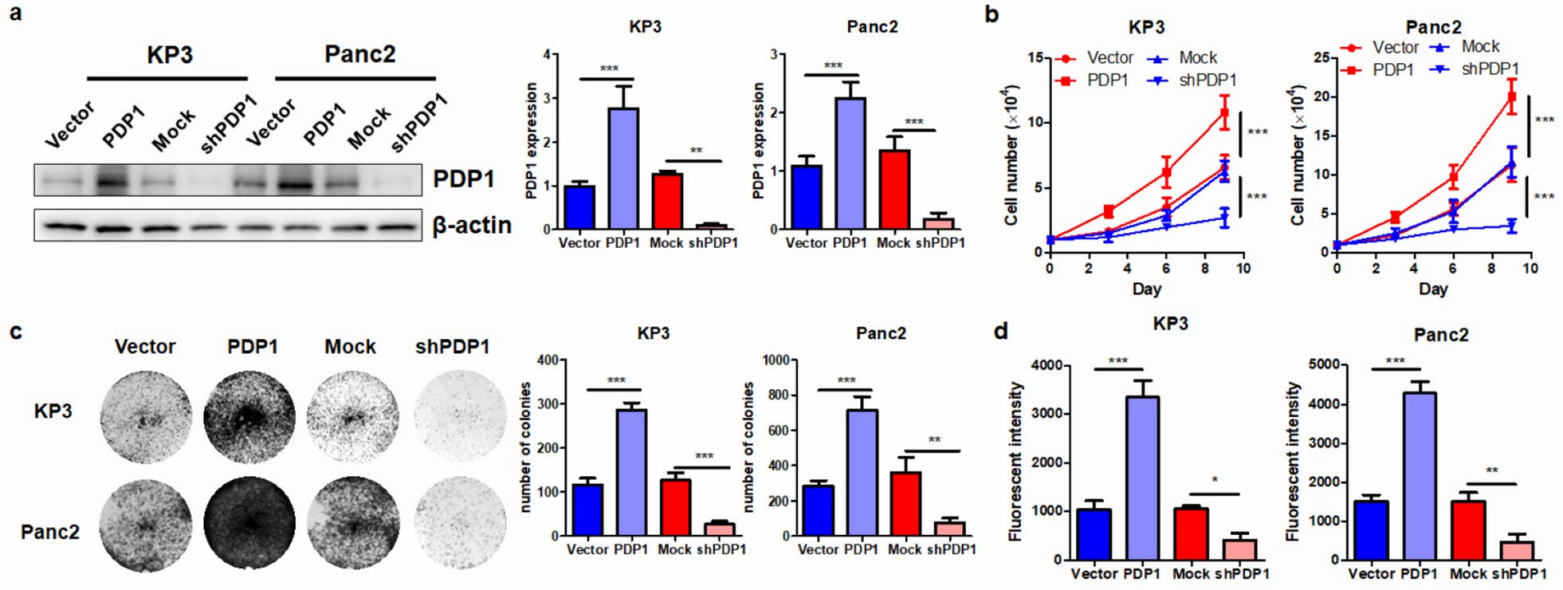
PDP1 regulated PDAC cell proliferation and migration in vitro. **a** Transfection of the PDP1 ORF clone or shRNA regulated endogenous expression of PDP1 in PDAC cells. **b** Overexpression of PDP1 promoted PDAC cell proliferation, while PDP1 suppressed PDAC cell proliferation. **c** Overexpression of PDP1 promoted colony formation of PDAC cells, while PDP1 suppressed PDAC cell colony number. **d** Overexpression of PDP1 promoted PDAC cell migration, while PDP1 suppressed PDAC cell migration. ***p < 0.001

To further elucidate the functional role of PDP1 in PDAC cells1, we first identified its in vitro function in a set of PDAC cells. The expression of PDP1 in different PDAC cells, including MiaPaca2, Panc1, Panc2, KP3, and Capan1, was screened to select suitable cell lines for functional studies (data not shown). Panc2 and KP3 were selected due to their moderate level of PDP1 expression among the cell lines. We then overexpressed PDP1 by transfecting a plasmid encoding the ORF of the genes while knocking down its expression by shRNA against PDP1 and established stably transfected cell lines by selection (Fig. 2a). A proliferation assay was performed to examine whether PDP1 expression could alter cell growth in PDAC. We found that in both cell lines, PDP1 overexpression significantly promoted the growth rate of the cells, while PDP1 knockdown had the opposite effects (Fig. 2b), suggesting that PDP1 expression may promote tumor cell proliferation in PDAC. This finding was further confirmed by colony formation assays, which showed that PDP1 overexpression potently increased the number of colonies formed by PDAC cells, while suppression of PDP1 reduced the ability to form colonies (Fig. 2c). In addition, as the in vitro invasiveness of tumor cells indicates the in vivo locoregional and distant spread of cancer, we examined the invasion of PDAC cell lines with a chamber assay. Overexpression of PDP1 significantly increased the number of cells invading through the extracellular matrix (Fig. 2d). These observations suggested that PDP1 expression could promote the in vitro proliferation and invasion of PDAC cells.

### PDP1 overexpression accelerated the in vivo tumor growth of PDAC

**Fig. 3:**
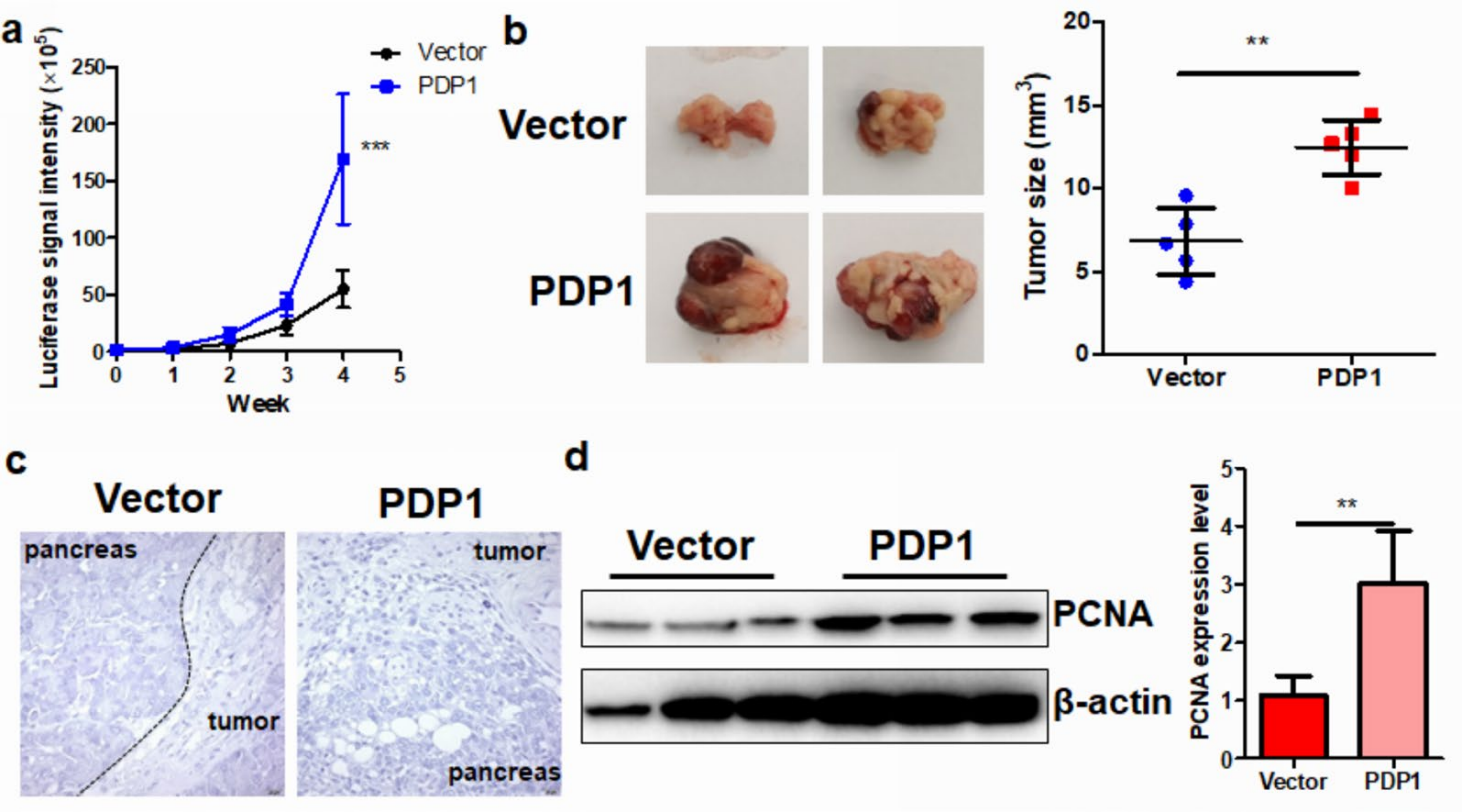
PDP1 overexpression promoted PDAC growth in vivo. **a** PDP1 overexpression significantly increased the luciferase signal in the orthotopic tumors of PDAC in mice; **b** PDP1 overexpression increased the tumor size of the orthotopic PDAC model; **c** H&E staining suggested that more cells were undergoing mitotic proliferation in the PDP1-overexpressing tumors than the control tumors; **d** PDP1-overexpressing tumors expressed a higher level of PCNA than the control tumors. ***p < 0.001

To further elucidate the in vivo functional role of PDP1, we established an orthotopic model of PDAC by injecting Panc2 cells into the pancreas of C57BL6/J mice. For noninvasive monitoring of tumor growth, Panc2 cells with or without PDP1 overexpression were stably infected with a luciferase reporter, and luciferin could be catalyzed to emit bioluminescent signals. Under an in vivo imager, we observed that overexpression of PDP1 could significantly increase the signal intensity of tumor cell-derived luciferase, indicating a larger tumor formed by PDP1-overexpressing PDAC cells than the control cells (Fig. 3a). End-point measurement of the dissected tumors showed that tumor growth was accelerated by PDP1 overexpression (Fig. 3b). Histological analysis by H&E staining showed that the PDP1-overexpressing PDAC cells had a more aggressive phenotype, e.g., an unclear border between pancreatic tissues and the tumor tissues, than the control cells (Fig. 3c). This result was further proven by the immunoblotting of PCNA, acellular marker of proliferation, in PDAC tissues; the expression was significantly higher in the PDP1-overexpressing PDAC tumors than the control tumors (Fig. 3d). These findings suggested that PDP1 overexpression could substantially accelerate the in vivo tumor growth of PDAC.

### PDP1 overexpression accelerated ATP-associated tumor cell growth in PDAC

**Fig. 4:**
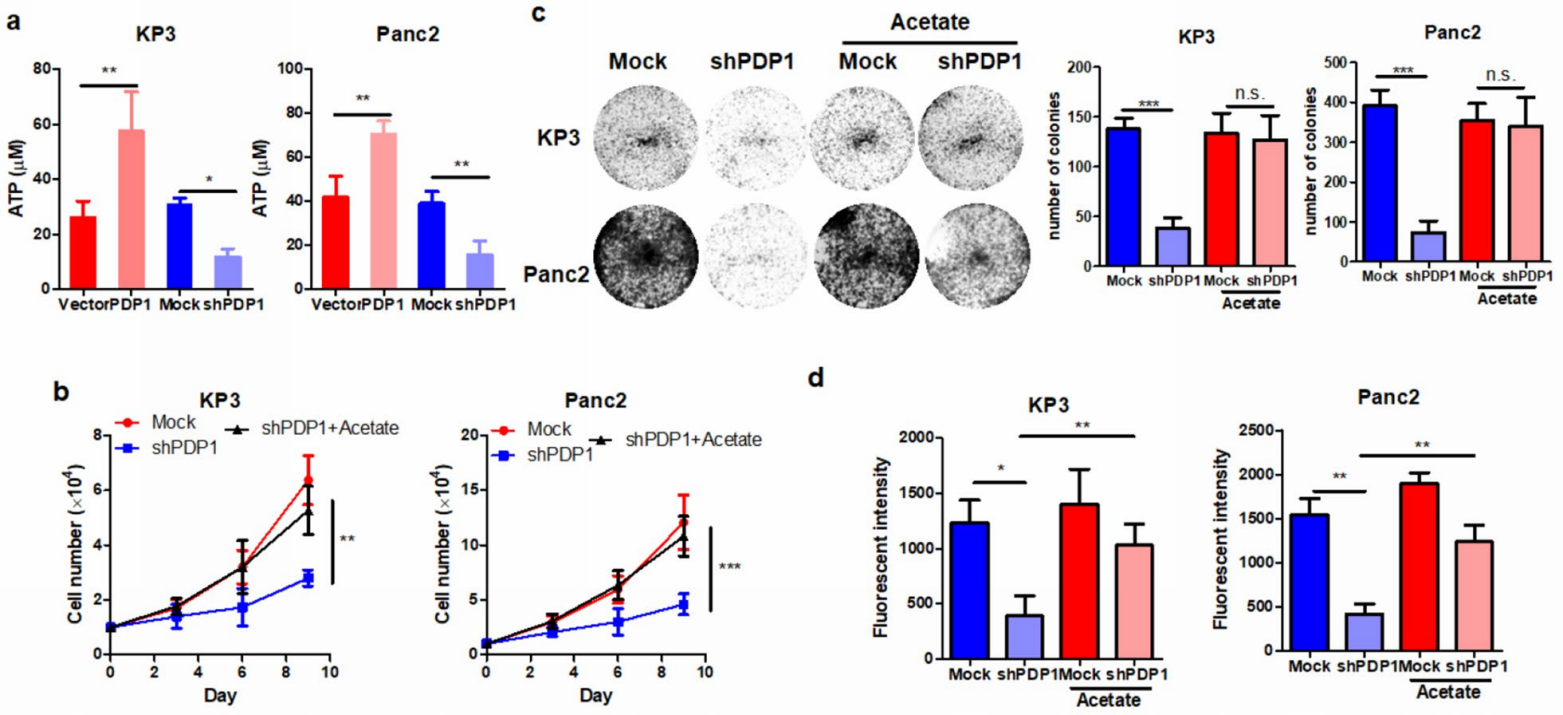
PDP1 induced PDAC cell proliferation and migration by inducing intracellular ATP. **a** PDP1 overexpression induced intracellular ATP content, while its knockdown reduced ATP levels. Supplementation of an alternative substrate of ATP production, acetate, restored cell proliferation (**b**), colony formation (**c**) and migration of PDP1 knockdown PDAC cells (**d**). ***p < 0.001

Mitochondrial PDP1 is an important regulator in energy production and metabolism in mammalian cells. PDP1 catalyzes the dephosphorylation and concomitant reactivation of the α subunit of the E1 component of the pyruvate dehydrogenase complex (PDC), whose activity is critical for energy metabolism through the TCA cycle and oxidative phosphorylation [11]. A previous study suggested that suppression of PDP1 expression in embryonic stem cells is associated with reduced production of ATP, the product of energy metabolism that is essential for many cellular processes [18]. Therefore, we examined the cellular content of ATP in PDAC cells. Overexpression of PDP1 could significantly increase the cellular ATP content in PDAC cells, while knockdown of the protein showed the opposite effects (Fig. 4a). To determine whether cellular ATP content plays an important role in mediating PDP1-induced cell proliferation, we supplied acetate as a substrate for ATP production by alternative mechanisms [19] in PDAC cells with PDP1 knockdown. The reconstitution of cellular ATP by acetate supplementation significantly reversed tumor cell proliferation in the cells with PDP1 knockdown (Fig. 4b). Colony formation and cell invasion assays proved that the suppressive effect of PDP1 knockdown was attenuated by ATP supplementation in PDAC cells (Fig. 4c, d). These observations suggested that PDP1-regulated cell proliferation and invasion in PDAC cells may be associated with the alteration of cellular ATP content and its related cellular activities.

### mTOR is a downstream signaling pathway that mediates PDP1-driven tumor cell proliferation in PDAC

**Fig. 5:**
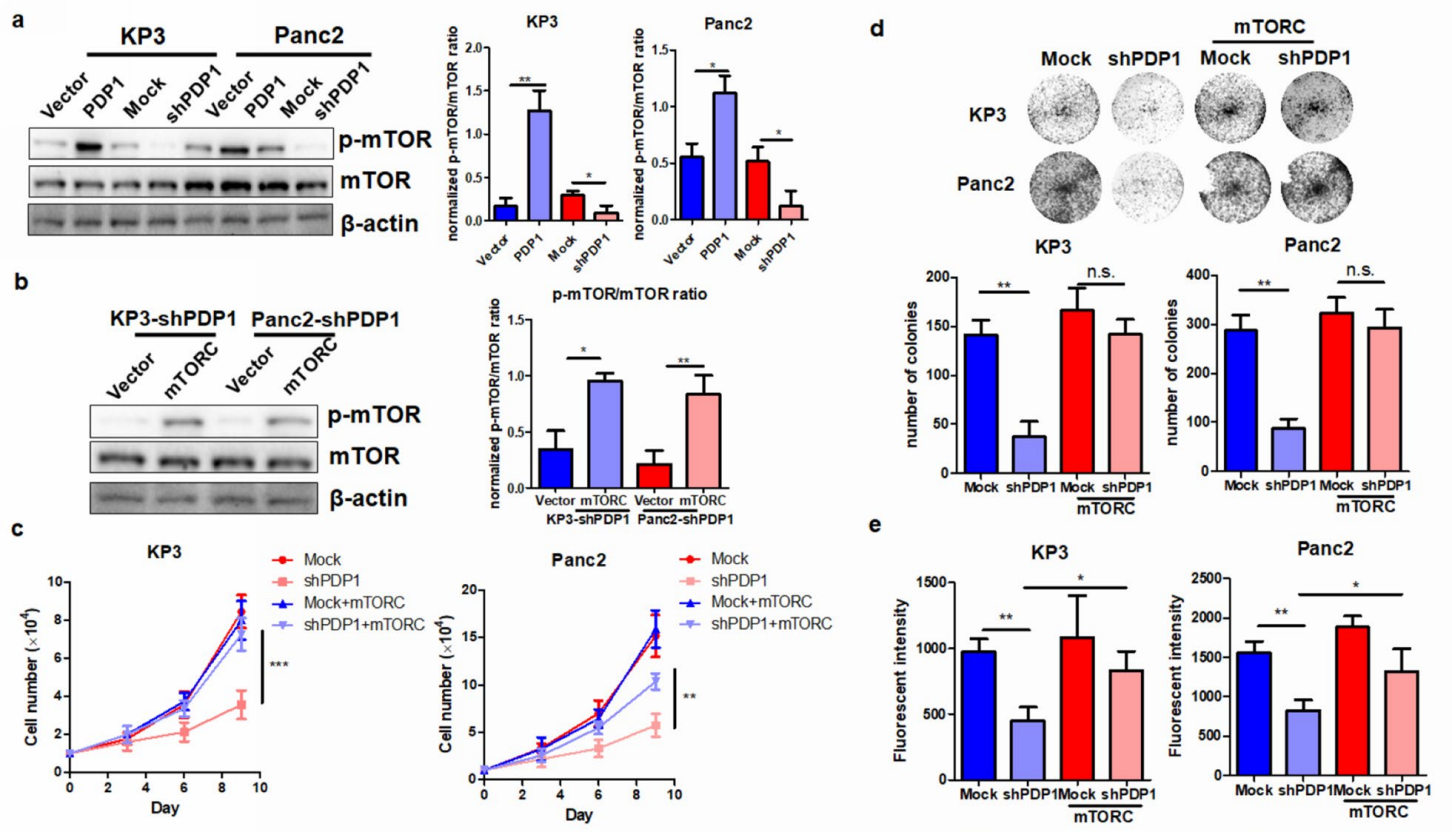
mTOR was responsible for PDP1-mediated PDAC cell proliferation and migration. **a** The phosphorylation of mTOR in PDAC cells, representing its activity, was measured by immunoblotting. PDP1 overexpression activated mTOR signaling, while its knockdown suppressed mTOR phosphorylation. **b** Transfection of constitutively activated mTORC reactivated mTOR signaling in PDP1 knockdown PDAC cells. mTORC represents a plasmid encoding a mutated mTOR with a change from arginine 2505 to proline, which leads to constitutive activation of mTOR regardless of intracellular signaling. Reactivation of mTOR restored cell proliferation (**c**), colony formation (**d**), and migration of PDP1 knockdown PDAC cells (**e**). ***p < 0.001

mTOR signaling is often aberrantly hyperactivated in various types of cancer cells in response to the abundant cellular ATP to promote tumor cell proliferation [20]. In our study, we examined the expression and activity of the mTOR pathway in PDAC cells with PDP1 knockdown. Upon knockdown of PDP1, mTOR phosphorylation was significantly downregulated without notable changes in total protein expression (Fig. 5a). To identify whether mTOR activity is crucial for PDP1-mediated tumor cell proliferation, we transfected a plasmid encoding the protein with a mutation that leads to constitutive activation of mTOR in PDAC cells with PDP1 knockdown (Fig. 5b). Constitutive activation of mTOR restored the proliferation rate in the PDAC cells with PDP1 knockdown (Fig. 5c), suggesting that mTOR activation is essential for PDP1-driven tumor cell growth in PDAC. In addition, reactivation of mTOR in PDAC cells with PDP1 knockdown reconstituted the capacity of PDAC cells to form colonies and pass through the ECM (Fig. 5d, e). These findings indicate that mTOR activation mediates PDP1-driven cell growth and invasion in PDAC as the downstream effector.

### AMPKα inhibition mediates PDP1-induced mTOR activation in PDAC cells

**Fig. 6:**
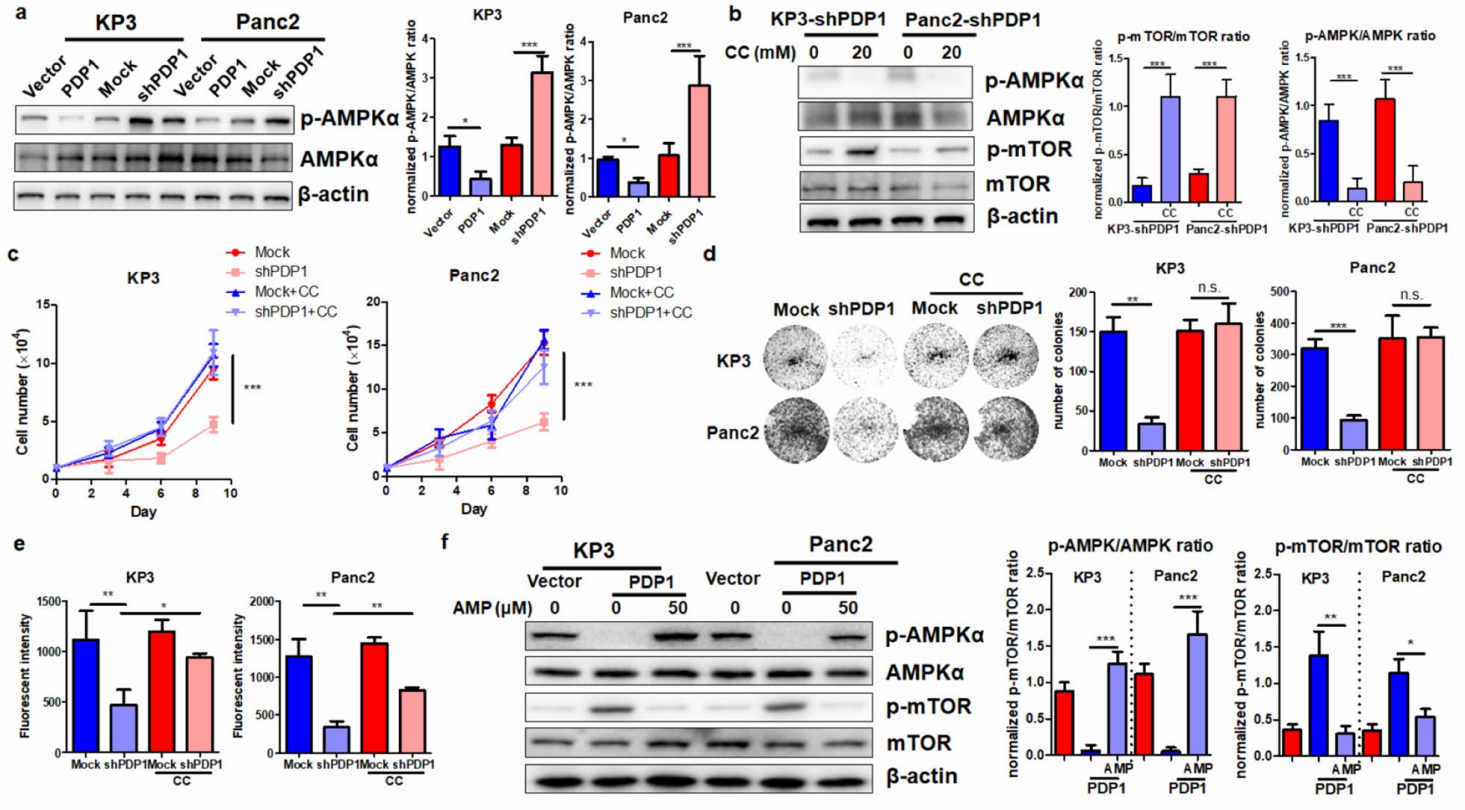
AMPK inactivation was responsible for PDP1-mediated PDAC cell proliferation and migration. **a** PDP1 overexpression suppressed AMPK signaling, while its knockdown induced activation via phosphorylation. **b** The presence of compound C (20 mM) inactivated AMPK signaling in PDP1 knockdown PDAC cells, which in turn reactivated mTOR signaling. Reactivation of mTOR restored cell proliferation (**c**), colony formation (**d**), and migration of PDP1 knockdown PDAC cells (**e**). **f** AMP was supplemented in the PDP1-overexpressing cells to adjust the intracellular AMP:ATP ratio. Increased AMP:ATP ratio in the PDP-overexpressing cells resulted in the restoration of AMPK activity but mTOR repression. ***p < 0.001

When the cellular content of ATP is insufficient, cells tend to activate AMPKα as a stress response pathway to limit growth-related signaling [21]. Activation of AMPKα in PDAC was found to restrict cancer cell proliferation and aggressiveness in previous studies [7, 22]. As PDP1 inhibition reduced ATP content, as we observed in the present study, we investigated whether AMPKα activity could be altered by PDP1. Overexpression of PDP1 potently suppressed the basal level of phosphorylated AMPKα without changing its total protein level, while repressing PDP1 could activate AMPKα (Fig. 6a). Inhibition of AMPKα in PDAC cells by its pharmacological inhibitor compound C (CC) restored mTOR activity (Fig. 6b), suggesting that AMPKα acts as the upstream regulator of mTOR in the PDAC cells with PDP1 knockdown. Furthermore, inhibition of AMPKα significantly reactivated the proliferation, colony formation, and invasion of PDAC cells (Fig. 6c–e). A previous study showed that an increased AMP:ATP ratio would activate AMPK activity [23]. We then added AMP to the PDP1-overexpressing cells and found that an increased AMP:ATP ratio led to the restoration of AMPK in the these cells (Fig. 6f). An opposite trend in mTOR activity was observed, as mTOR is a downstream effector of AMPK. These observations, together with the other findings in this study, indicated that PDP1 regulates PDAC growth by modulating the ATP/AMPKα/mTOR pathway.

## Discussion

**Fig. 7:**
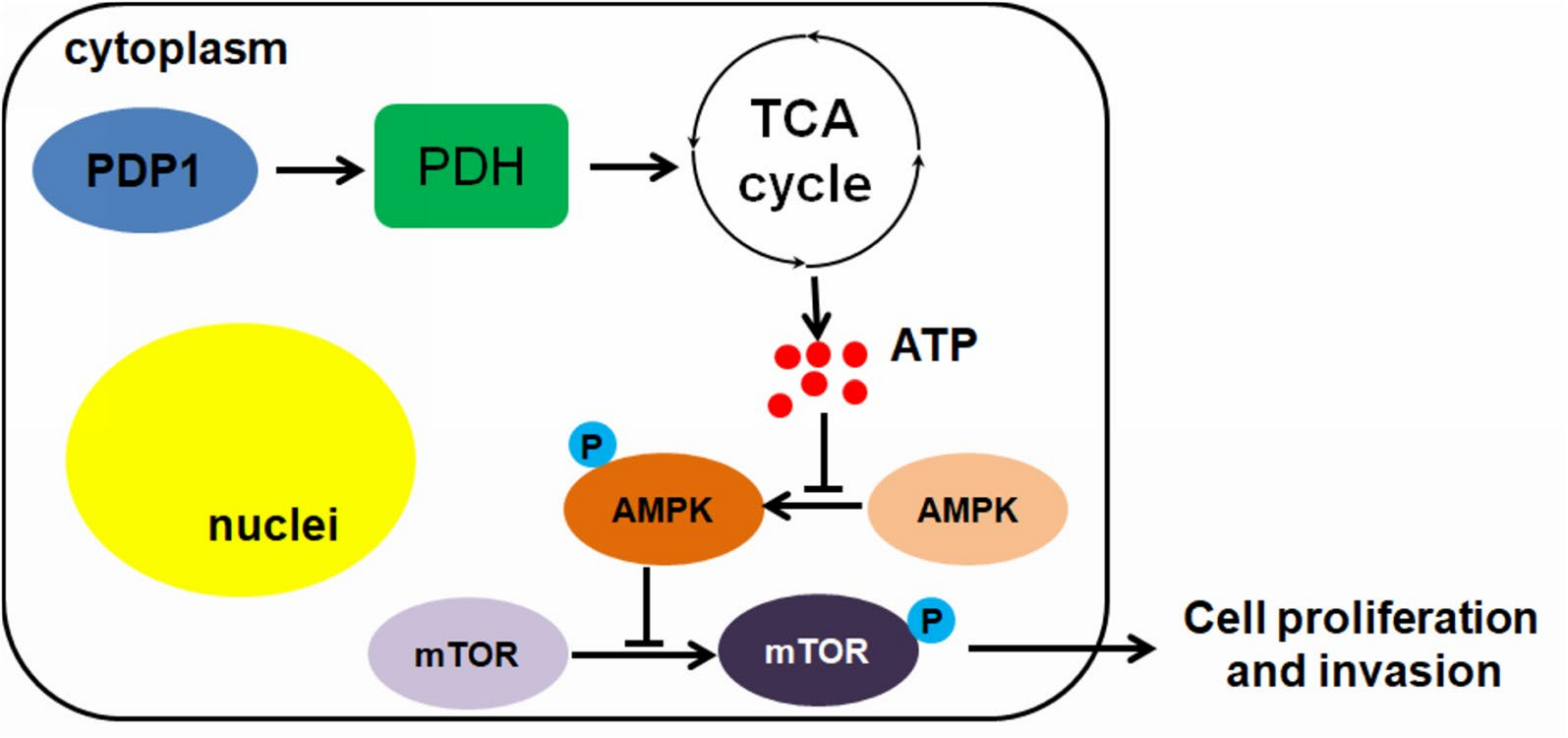
Schematic regulation of PDP1 in PDAC. Overexpression of PDP1 may activate PDH, which in turn accelerates the TCA cycle, which provides reducing equivalents in the form of NADH, FADH2, etc. for the ATP production by oxidative phosphorylation. The abundance of cellular ATP inhibits AMPK activation, restoring the mTOR signaling that can promote proliferation and invasion of PDAC cells

In this study, we observed that PDP1 was overexpressed in human PDAC. Overexpression of PDP1 in PDAC cells promoted cell proliferation and migration in vitro and stimulated tumor growth in a murine model of PDAC, which could be related to the increase in intracellular energy production (Fig. 7). Expression of PDP1 was reported to be associated with ATP generation in previous studies; for instance, PDP1 expression and activity were shown to contribute to a higher aerobic capacity of the muscle, suggesting a sustainable level of oxidative phosphorylation-related ATP production [24]. Another study also suggested that activation of PDP1 in cancer cells could induce a switch from cellular glycolysis to oxidative phosphorylation, the more efficient method of ATP production [24]. The depletion of cellular ATP abolished PDP1-induced PDAC cell proliferation, migration, and invasion, suggesting that ATP generation was the essential step by which PDP1 triggers these cellular processes. PDP1 expression was also found to be critical in some other cellular processes, such as recovery from injury and cell differentiation [18, 25]. Given the important regulatory role of PDP1 in the physiological function of PDC, our observations may suggest that overexpression of PDP1 in PDAC cells functions to hyperactivate the PDC system, overloading the cellular substrate of the tricarboxylic acid cycle, which in turn accelerates the oxidative phosphorylation process that can generate more ATP essential for cellular processes.

We used acetate as an alternative carbon source in tumor cells with PDP1 knockdown. PDP1 is a critical enzyme involved in the pathway of ATP production by consuming glucose. PDP1 is involved the reaction of pyruvate to acetyl-CoA, the direct source of the TCA cycle. This facilitates the production of reducing equivalents in the form of NADH, FADH2, etc. by TCA cycles, which can be used by oxidative phosphorylation for the production of ATP. Acetate has been reported as an additional source for ATP production in cancer cells, especially when glucose/glutamine is depleted [26]. The direct ligation of acetate to CoA by acetyl-CoA synthetases can yield acetyl-CoA in the TCA cycle [27]. The TCA cycle can, therefore, produce reducing equivalents in the form of NADH, FADH2, etc., which were used for ATP production by oxidative phosphorylation in PDP1 knockdown cells. As we assumed that suppression of ATP production in PDP1 knockdown cells is responsible for inhibition of tumor growth, we used acetate as an alternative source of ATP production in these cells, and our observations proved that ATP restoration in PDP1 knockdown cells recovered tumor cell proliferation and growth, supporting our claims. We also observed that the addition of acetate had a minimal effect on tumor growth in mock tumor cells. Mock cells can consume glucose to produce acetate, which is then directly ligated by acetyl-CoA synthetases in the TCA cycle. The reason for these results after addition of acetate to cells with an intact pathway of glucose metabolism is not fully understood; however, as the TCA cycle has a strict regulatory mechanism that involves production of reducing equivalents as sources of ATP production, this complicated process has rate-limiting steps even when the acetyl-CoA amount is overwhelming. In this regard, the addition of acetate may not further accelerate cell proliferation and growth in mock cells, which can normally consume glucose to produce ATP.

We observed that AMPK/mTOR signaling was involved in PDP1 regulation of PDAC progression. As an energy sensor, AMPK is activated when the intracellular ATP level is insufficient and triggers a series of downstream responses that inhibit rapid cell proliferation; hence, AMPK has been frequently identified as a potential target in anticancer treatment [28]. PDP1 overexpression could induce activation of PDC, which in turn generates more substrate for the production of ATP. In this case, overexpression of PDP1 in our study resulted in sustained repression of AMPK signaling due to the abundance of intracellular ATP that led to suppression of multiple downstream proliferation-associated mechanisms. mTOR is a key molecule that mediates rapid protein synthesis during cancer progression [29]. Phosphorylation of mTOR signaling activates translational control by initiating the formation of the ribosome complex as well as the association of mRNA at its 5’-cap [30]. This process was halted under nutrient-deprived conditions, in which mTOR was dephosphorylated and could not initiate cap-dependent translation [31]. The activation of AMPK as a nutrient sensor in some previous studies has been found to primarily downregulate mTOR activity in cancers [32, 33]. PDP1-mediated AMPK repression therefore could be a possible mechanism to facilitate mTOR-associated PDAC progression, which was confirmed by our observation that suppression of AMPK in PDP1 knockdown cells revoked mTOR signaling.

## Conclusion

In summary, in this study, we identified the expression pattern and function of PDP1 in PDAC. PDP1 was overexpressed in PDAC samples compared with normal pancreatic samples, and high expression of PDP1 was correlated with a poor prognosis of PDAC patients. Overexpression of PDP1 promoted the in vitro proliferation, colony formation, and migration of PDAC cells, while knockdown of PDP1 had the opposite effects. Overexpression of PDP1 stimulated the in vivo growth of orthotopic PDAC tumors in a murine model. PDP1 regulated the production of intracellular ATP, and supplementation with ATP recovered tumor cell proliferation and migration in PDP1 knockdown PDAC cells. This result could be related to the alteration of mTOR activity in PDAC cells, and recovery of mTOR activity in PDP1 knockdown PDAC cells restored cell proliferation and migration. Mechanistically, PDP1 knockdown activated AMPK signaling, which in turn suppressed mTOR activity. Repression of AMPK in PDP1 knockdown cells restored the proliferation and migration of PDAC. Our study suggested that PDP1 may be an attractive target for the diagnosis and treatment of PDAC.

## Data Availability

The datasets used and/or analysed during the current study are available from the corresponding author on reasonable request.

## References

[1] Collaborators GBDPC (2019). The global, regional, and national burden of pancreatic cancer and its attributable risk factors in 195 countries and territories, 1990–2017: a systematic analysis for the Global Burden of Disease Study 2017. Lancet Gastroenterol Hepatol.

[2] Rahib L, Smith BD, Aizenberg R, Rosenzweig AB, Fleshman JM, Matrisian LM (2014). Projecting cancer incidence and deaths to 2030: the unexpected burden of thyroid, liver, and pancreas cancers in the United States. Cancer Res.

[3] Kim J, Bamlet WR, Oberg AL, Chaffee KG, Donahue G, Cao XJ, Chari S, Garcia BA, Petersen GM, Zaret KS (2017). Detection of early pancreatic ductal adenocarcinoma with thrombospondin-2 and CA19-9 blood markers. Sci Transl Med.

[4] Hackert T (2018). Surgery for Pancreatic Cancer after neoadjuvant treatment. Ann Gastroenterol Surg.

[5] Satoi S, Yamamoto T, Yamaki S, Sakaguchi T, Sekimoto M (2020). Surgical indication for and desirable outcomes of conversion surgery in patients with initially unresectable pancreatic ductal adenocarcinoma. Ann Gastroenterol Surg.

[6] Chandana S, Babiker HM, Mahadevan D (2019). Therapeutic trends in pancreatic ductal adenocarcinoma (PDAC). Expert Opin Investig Drugs.

[7] Chen K, Qian W, Li J, Jiang Z, Cheng L, Yan B, Cao J, Sun L, Zhou C, Lei M (2017). Loss of AMPK activation promotes the invasion and metastasis of pancreatic cancer through an HSF1-dependent pathway. Mol Oncol.

[8] Maj MC, Cameron JM, Robinson BH (2006). Pyruvate dehydrogenase phosphatase deficiency: orphan disease or an under-diagnosed condition?. Mol Cell Endocrinol.

[9] Stacpoole PW (2017). Therapeutic targeting of the pyruvate dehydrogenase complex/pyruvate dehydrogenase kinase (PDC/PDK) axis in cancer. J Natl Cancer Inst.

[10] Chen J, Guccini I, Di Mitri D, Brina D, Revandkar A, Sarti M, Pasquini E, Alajati A, Pinton S, Losa M (2018). Compartmentalized activities of the pyruvate dehydrogenase complex sustain lipogenesis in prostate cancer. Nat Genet.

[11] Bedoyan JK, Hecht L, Zhang S, Tarrant S, Bergin A, Demirbas D, Yang E, Shin HK, Grahame GJ, DeBrosse SD (2019). A novel null mutation in the pyruvate dehydrogenase phosphatase catalytic subunit gene (PDP1) causing pyruvate dehydrogenase complex deficiency. JIMD Rep.

[12] Cameron JM, Maj MC, Levandovskiy V, MacKay N, Shelton GD, Robinson BH (2007). Identification of a canine model of pyruvate dehydrogenase phosphatase 1 deficiency. Mol Genet Metab.

[13] Cameron JM, Maj M, Levandovskiy V, Barnett CP, Blaser S, Mackay N, Raiman J, Feigenbaum A, Schulze A, Robinson BH (2009). Pyruvate dehydrogenase phosphatase 1 (PDP1) null mutation produces a lethal infantile phenotype. Hum Genet.

[14] Leblanc PJ, Mulligan M, Antolic A, Macpherson L, Inglis JG, Martin D, Roy BD, Peters SJ (2008). Skeletal muscle type comparison of pyruvate dehydrogenase phosphatase activity and isoform expression: effects of obesity and endurance training. Am J Physiol Regul Integr Comp Physiol.

[15] Huang B, Wu P, Popov KM, Harris RA (2003). Starvation and diabetes reduce the amount of pyruvate dehydrogenase phosphatase in rat heart and kidney. Diabetes.

[16] Piccinini M, Mostert M, Alberto G, Ramondetti C, Novi RF, Dalmasso P, Rinaudo MT (2005). Down-regulation of pyruvate dehydrogenase phosphatase in obese subjects is a defect that signals insulin resistance. Obes Res.

[17] Shan C, Kang HB, Elf S, Xie J, Gu TL, Aguiar M, Lonning S, Hitosugi T, Chung TW, Arellano M (2014). Tyr-94 phosphorylation inhibits pyruvate dehydrogenase phosphatase 1 and promotes tumor growth. J Biol Chem.

[18] Heo HJ, Kim HK, Youm JB, Cho SW, Song IS, Lee SY, Ko TH, Kim N, Ko KS, Rhee BD (2016). Mitochondrial pyruvate dehydrogenase phosphatase 1 regulates the early differentiation of cardiomyocytes from mouse embryonic stem cells. Exp Mol Med.

[19] Liu X, Cooper DE, Cluntun AA, Warmoes MO, Zhao S, Reid MA, Liu J, Lund PJ, Lopes M, Garcia BA (2018). Acetate production from glucose and coupling to mitochondrial metabolism in mammals. Cell.

[20] Jones AT, Yang J, Narov K, Henske EP, Sampson JR, Shen MH (2019). Allosteric and ATP-competitive inhibitors of mtor effectively suppress tumor progression-associated epithelial-mesenchymal transition in the kidneys of Tsc2(±) mice. Neoplasia.

[21] Liu G, Kuang S, Cao R, Wang J, Peng Q, Sun C (2019). Sorafenib kills liver cancer cells by disrupting SCD1-mediated synthesis of monounsaturated fatty acids via the ATP-AMPK-mTOR-SREBP1 signaling pathway. FASEB J.

[22] Duan W, Chen K, Jiang Z, Chen X, Sun L, Li J, Lei J, Xu Q, Ma J, Li X (2017). Desmoplasia suppression by metformin-mediated AMPK activation inhibits pancreatic cancer progression. Cancer Lett.

[23] Hardie DG, Alessi DR (2013). LKB1 and AMPK and the cancer-metabolism link—ten years after. BMC Biol.

[24] Love LK, LeBlanc PJ, Inglis JG, Bradley NS, Choptiany J, Heigenhauser GJ, Peters SJ (2011). The relationship between human skeletal muscle pyruvate dehydrogenase phosphatase activity and muscle aerobic capacity. J Appl Physiol (1985)..

[25] Xing G, Ren M, O’Neill JT, Sharma P, Verma A, Watson WD (2012). Pyruvate dehydrogenase phosphatase1 mRNA expression is divergently and dynamically regulated between rat cerebral cortex, hippocampus and thalamus after traumatic brain injury: a potential biomarker of TBI-induced hyper- and hypo-glycaemia and neuronal vulnerability. Neurosci Lett.

[26] Schug ZT, Peck B, Jones DT, Zhang Q, Grosskurth S, Alam IS, Goodwin LM, Smethurst E, Mason S, Blyth K (2015). Acetyl-CoA synthetase 2 promotes acetate utilization and maintains cancer cell growth under metabolic stress. Cancer Cell.

[27] Corbet C, Feron O (2015). Metabolic and mind shifts: from glucose to glutamine and acetate addictions in cancer. Curr Opin Clin Nutr Metab Care.

[28] Jiang X, Tan HY, Teng S, Chan YT, Wang D, Wang N (2019). The role of AMP-activated protein kinase as a potential target of treatment of hepatocellular carcinoma. Cancers (Basel).

[29] Forester CM, Zhao Q, Phillips NJ, Urisman A, Chalkley RJ, Oses-Prieto JA, Zhang L, Ruggero D, Burlingame AL (2018). Revealing nascent proteomics in signaling pathways and cell differentiation. Proc Natl Acad Sci U S A.

[30] Klann K, Tascher G, Munch C (2020). Functional translatome proteomics reveal converging and dose-dependent regulation by mTORC1 and eIF2alpha. Mol Cell.

[31] Sabatini DM (2006). mTOR and cancer: insights into a complex relationship. Nat Rev Cancer.

[32] Leibovitch M, Topisirovic I (2018). Dysregulation of mRNA translation and energy metabolism in cancer. Adv Biol Regul.

[33] Kim J, Kundu M, Viollet B, Guan KL (2011). AMPK and mTOR regulate autophagy through direct phosphorylation of Ulk1. Nat Cell Biol.

